# Minimizing the Forces in the Single Point Incremental Forming Process of Polymeric Materials Using Taguchi Design of Experiments and Analysis of Variance

**DOI:** 10.3390/ma15186453

**Published:** 2022-09-17

**Authors:** Nicolae Rosca, Tomasz Trzepieciński, Valentin Oleksik

**Affiliations:** 1 Faculty of Engineering, “Lucian Blaga”, University of Sibiu, Victoriei Bd. 10, 550024 Sibiu, Romania; 2 Department of Manufacturing and Production Engineering, Faculty of Mechanical Engineering and Aeronautics, Rzeszow University of Technology, al. Powst. Warszawy 8, 35-959 Rzeszów, Poland

**Keywords:** polymer incremental forming, polyamide, polyethylene, statistical analysis, force minimizing

## Abstract

The aim of the present paper is that of conducting a study on the basis of which the optimal parameters for the manufacturing of polymer parts by means of the single point incremental forming process can be chosen in such a way that the process forces have minimum values. Two polymeric materials with a 3 mm thickness, polyamide and polyethylene, were chosen for the analysis. The other input parameters that were considered were: the punch diameter, the step on vertical direction and the wall angle. The Taguchi method was chosen for the design of experiments. Each of the input parameters, except for the material, were varied on three levels—for the punch diameter: 6 mm, 8 mm and 10 mm; for the step on vertical direction: 0.5 mm, 0.75 mm and 1 mm; and for the wall angle: 50°, 55° and 60°. Forces were measured in the three directions of the coordinate axes and the results were analyzed based on the signal-to-noise ratio and an analysis of variance with the aim of minimizing the values of the forces. Considering the input parameters analyzed, it was concluded that the forces are most influenced by the material, followed by the punch diameter, the step on vertical direction and the wall angle.

## 1. Introduction

The single point incremental forming process has gained a reputation among forming processes over the last 20–30 years due to its advantages over other forming processes: the use of simple, modular dies which are much cheaper than the ones used in deep-drawing, the possibility of using computer numerical control (CNC) machine tools [1] or industrial robots [2] instead of presses, the possibility of obtaining parts with complex geometries even when limited to simple dies and, last but not least, the ability to obtain drastically reduced forming forces due to the progressive deformation of the material. However, the single point incremental forming process has its limitations as well, the most significant of which being the long manufacturing time, which leads to this process only being used for parts manufactured in small batches or one-off productions. Initially, the most commonly manufactured materials were metallic materials (steel, aluminum, copper, titanium and their alloys [3]), but more recently, this forming process has also found its application in polymeric materials. The first research on single point incremental forming (SPIF) of polymers was published in the early 2000s, and a first state-of-the-art synthesis in this field was published by Martins and his co-authors in 2009 [4]. Two other recent review papers have studied the latest advances in the incremental forming of polymer parts, ranging from room temperature and high temperature processing to single point or two point incremental forming [5,6]. Incremental forming has been used to manufacture parts from various polymeric materials: polyvinylchloride sheets [7], polycarbonate sheets [8], polypropylene sheets [9] and even shape memory polymer foam [10]. Special attention has been paid to the manufacturing of polymer composite parts. Thus, Ambrogio et al. [11] presented a study on the SPIF processing of a composite material made of a polyamide matrix in which short glass fibers were melted. The amount of short glass fibers in the volume of the composite material was 12%. The study focused on determining the accuracy of the deformed parts, as well as on a microscopic analysis of the material following the forming process. Another study investigated the possibility of making SPIF-processed cranial implants from a reinforced material made of polymethyl methacrylate (PMMA) [12]. Most papers related to the incremental forming of composite materials focused on polyamides with different reinforcing elements, such as: nanocomposites with montmorillonite (MMT) filler clay [13] and glass fibers in amounts of up to 30 wt.% [14]. Emani et al. presented a paper in which they analyzed methods of improving the SPIF formability of glass-fiber-reinforced polyamide composite materials [15]. They concluded that the SPIF formability of these materials increased with increasing forming temperature and decreasing fraction of glass fibers from the total volume of the workpiece. Another material manufactured with SPIF, in order to increase the rigidity, was paperboard with different fiber orientations (0°, 45° and 90°) and different moisture content (4.5%, 10% and 20%) [16]. The applications of polymer parts manufactured by SPIF are diverse and range from manufacturing parts for the aerospace industry [17], given the weight reduction while maintaining sufficient strength, to applications related to implant manufacturing [18], due to the fact that many of them are biocompatible materials. Centeno et al. [19] reported on the methodology for producing custom cranial implants, starting with computerized tomography and ending with the SPIF fabrication of polymeric materials, specifically a polyvinylchloride sheet and two polycarbonate sheets. In the study, the method of eliminating springback was also presented for the development of the punch trajectory, in order to achieve high accuracy. Another study analyzed the formability of polymeric materials during SPIF, both at room temperature and at elevated temperatures [20]. Experimental analysis of the failure modes of polycarbonate sheets was investigated by Rosa-Sainz et al. [7]. Experimental research related to plastic strains and failure modes of polyvinylchloride sheets [21] and polypropylene sheets [22] has contributed to understanding the deformation mechanism, as well as to estimating the formability of castor polymeric materials. Another review article summarized the formability of different polymer matrix composites [23]. The incremental forming process can be performed either in single point or with support from the opposite side of the punch. Most of the publications in the field of polymeric materials deal with the SPIF process, but there are also papers addressing the two point incremental forming [24,25]. In the case of parts made of polymeric materials and manufactured by incremental forming, the main defects that occurred were those caused by springback [26], wrinkle defects [27] and twist defects [28]. While springback and wrinkle defects are also common to the SPIF processing of other types of materials, the twist defects are more specific to polymeric materials that have a lower rigidity and twist during the forming around the vertical axis of the part. Yang and Chan investigated the twist phenomena occurrence mechanism in the SPIF of polyvinylchloride sheets [28]. They calculated the twist angle based on the law of conservation of energy. In order to increase the accuracy of thermoplastic parts processed by SPIF, Maas et al. proposed the overbending of the workpiece, with the aim of reducing the springback [29]. One phenomenon that occurs in the SPIF manufacturing of polymeric materials is that of self-heating due to the frictional forces between the punch and the polymer sheet. Formisano et al. studied this phenomenon and attempted to control it by the rotational speed of the punch, in such a way that the phenomenon improved the formability of the process [30]. A comparative analysis of the behavior of polymeric materials during incremental forming at elevated and room temperatures was performed by Kulkarni and Mocko [31]. Hot incremental forming of glass-fiber-reinforced polymers [32] or biocomposite materials developed from thermoplastic matrix [33,34] has been reviewed in recently published papers. A synthesis of the hot incremental forming process of polycarbonate sheets was published by Sridhar and Rajenthirakumar [35], addressing issues related to geometrical accuracy, thickness distribution and fracture formation. A number of published studies deal with the finite element method applied for the numerical simulation of the SPIF processing of polymeric materials [36,37]. These studies present different models: a thermomechanical coupled model [36], and a viscoplasticity based on overstress model [37]. Other studies not only perform numerical simulations, but also validate them by experimental investigations [38,39]. Another concern related to polymeric materials but also to the SPIF process is that of forming sandwich materials (steel/polymer/steel) [40]. The tools for the SPIF processing of polymeric materials can be made by classical cutting processes or by new, innovative methods such as rapid prototyping. A new method to produce the tools needed for the incremental forming process of polymeric materials, named direct rapid tooling, was presented by Afonso et al. [41]. Over the years, the SPIF process parameters for the manufacturing of polymeric materials have been analyzed in a number of papers. For instance, the influence of the punch diameter, wall-angle of the part, step on vertical direction and feed rate on the springback, thickness reduction and surface roughness for polypropylene sheets was studied by Jain et al. [42]. Another study examined the influence of the wall-angle of the part, step on vertical direction and feed rate on the same output parameters, but for polyvinylchloride sheets [43]. Thangavel et al. [44] studied the influence of the roller ball diameter, step on vertical direction, feed rate, initial sheet thickness and spindle speed during single point incremental forming with rolling friction for four polymeric materials: high density polyethylene, polycarbonate, polyvinylchloride and polypropylene. Formability (maximum depth before failure) and thickness distribution were chosen as response functions. Davarpanah et al. evaluated the effect of the vertical step and spindle speed on the failure modes and microstructural properties of polymeric materials formed with SPIF [45]. A first paper analyzing the forces during the SPIF process of polymeric materials is that of Bagudanch et al. [46]. They studied the influence of the vertical step, spindle speed, feed rate, punch diameter and initial sheet thickness on the maximum forming force during the SPIF of polyvinylchloride sheets. In addition to the maximum force, the maximum depth before failure and surface roughness were also chosen as output parameters. Considering the spindle feed, they also analyzed the self-heating process caused by the frictional forces occurring in the incremental forming process. Another work allows the prediction of forces in SPIF, based on the contact area and friction coefficient [47]. The analytical results were experimentally verified for two materials, polycarbonate and polyvinylchloride sheets. A recent paper presents the friction stir incremental forming process for polyoxymethylene sheets [48]. This study also presents the influence of the punch diameter, step on vertical direction, spindle speed and feed rate on the maximum force, temperature obtained as a result of the self-heating process and maximum depth before failure.

The present paper studies the single point incremental forming process applied to polymeric materials. As is well known, the process was developed as a result of economic necessity, as conventional processes led to high costs that could not be recovered in the cases of small series and one-off productions. Moreover, the paper is dedicated to the application of this technology to polymeric materials, which are low-density materials, increasingly used in the automotive industry, medical device industry, electrotechnics industry and so on, having been less studied than metallic materials. The impact of the results obtained in this study should not only be considered from a technical, engineering point of view, but from a much greater one, leading to economic or even environmental benefits, as confirmed by a recently published study [49]. This is also confirmed by previous studies analyzing the parameters influencing energy consumption in polymer processing or another study, also focusing on energy consumption, concerning the possibility of reusing waste through incremental forming [50]. Two other recent studies analyzed the energy consumption, carbon emissions and product cost in the context of the sustainability of the incremental forming process [51,52]. The environmental impact of the incremental forming process has also been analyzed from the life cycle assessment perspective [53]. Although the present study did not intend to analyze the incremental forming process with regard to these aspects, it nevertheless makes an essential contribution in this direction, by identifying ways of reducing the forces in the process and thus the amount of energy consumed.

All reviewed papers only study the force on vertical direction (on Oz direction) but not the other two horizontal components (on Ox and Oy direction), which are also very important in terms of the stress applied to the machine used in SPIF. Moreover, there is a lack of knowledge concerning the way in which the SPIF process parameters (step size, punch diameter and wall angle) affect the horizontal components of the forming force. This matter is of particular importance when forming polymeric materials that exhibit a formability that is different to that of metallic sheets commonly tested in incremental sheet forming. A forming tool mounted in a gripper of the shelf-mounted robot was used in the investigations. Achieving the lowest possible forming forces is also important in terms of minimizing the elastic deformation of the robot arms, which determine the forming accuracy. As can be seen from the above, although there are papers dealing with the issue of force variation during the SPIF process and the influence of various process parameters on the maximum force, the purpose of the present paper is, by means of the Taguchi design of experiments and an analysis of variance (ANOVA), to establish the influence of certain essential parameters, such as the punch diameter, step on vertical direction, wall angle and material type, on all forces in the process. The aim of the study is therefore to find the optimal variants of the input parameters that lead to the minimization of the forces in the process. Moreover, for the two chosen materials, specifically polyamide and polyethylene, the regression relations for the three categories of forces were also obtained. To achieve the purpose of the present research, experiments were carried out to form the two polymeric sheets (polyamide and polyethylene) by using the shelf-mounted Kuka Kr210-2 robot. The input parameters considered were the step size, the punch diameter and the wall angle. The Taguchi method was used to find the minimum number of experiments to be performed within the permissible limit of factors and levels. These SPIF parameters were varied on three levels—for the punch diameter: 6 mm, 8 mm and 10 mm; for the step on vertical direction: 0.5 mm, 0.75 mm and 1 mm; and for the wall angle: 50°, 55° and 60°. The PCB 261A13 force sensor was used to measure the two horizontal components of the forming force that act in the workpiece plane and the axial component in the direction of the forming tool. The results were analyzed based on the signal-to-noise (S/N) ratio and an analysis of variance. In order to obtain the mathematical relationships between the forming force components in SPIF and the input parameters, a regression analysis was performed.

Apart from the materials studied in the paper, namely polyamide, polyethylene and polytetrafluoroethylene, the behavior of other polymeric materials regarding the incremental forming process at room temperature, such as polyoxymethylene and polyvinylchloride, was also initially studied. Due to the fact that these materials developed fractures in the initial stages of the incremental forming process, it was decided not to include the respective materials in the present study.

## 2. Materials and Methods

The experimental research in the paper is structured in two directions: research quantifying the formability of polymeric materials and research strictly related to the incremental forming process of these materials. The research quantifying the formability of the materials is performed by uniaxial tensile testing of polymeric materials.

### 2.1. Uniaxial Tensile Testing of Polymeric Materials

Tensile testing always provides clues regarding the plastic deformation behavior of a material. Thus, in order to identify which polymeric materials are best subjected to deformation by incremental process, three polymeric materials were chosen: polyamide 6.6 (PA), high density polyethylene (HDPE) and polytetrafluoroethylene (PTFE).

An Instron 5587 tensile testing machine (Norwood, MA, USA) was used for the tests. The drive system of the Instron 5587 machine is electromechanical and the movable crossbar is guided by means of a ball screw guide system. The main features of the testing machine are: maximum load cell capacity of 300 kN, maximum test speed of 500 mm/min, load cell with a linearity of ±0.25% and repeatability of ±0.25% for readings taken at 0.4–100% of the maximum load cell capacity. The machine is connected to a computer via an Ethernet interface. Calibration and testing operations are controlled via the Bluehill 2.0 software package which comes with the machine and is installed on the computer. The software package allows the selection of the specimen type (standard ISO or ASTM types), specimen size, test speed, as well as the means by which specimen breakage is detected. The Bluehill 2.0 software package also monitors the testing, generates measurement reports and performs statistical processing of the data.

A 3 mm thickness was used for all three polymer materials. The calibrated area of the specimens had an initial width of 20 mm and length of 120 mm. To perform the tests sets of 5 specimens were made for each material. There are several methods by which the specimens can be cut: mechanical die cutting, laser cutting and waterjet cutting. Due to the specificity of the materials used, waterjet cutting was chosen as cutting method, on the one hand because laser cutting would not be viable, as the material is flammable, and on the other hand because cutting on a die would produce a significant burr due to the high elongation present in these types of materials.

After making the specimens, the settings for the actual test were defined in the testing machine’s built-in program, Bluehill. A test speed of 20 mm/min was chosen, in accordance with current standards, and the maximum elongation was set to 700%, so as not to exceed the machine’s limits. The following output data were chosen: the longitudinal modulus of elasticity (E) in megapascals (MPa), yield stress (Ys) in MPa, the maximum stress in MPa, and the strain corresponding to the maximum stress in %.

### 2.2. Experimental Layout for the Study of the Behavior of Polymeric Materials during Single Point Incremental Forming

**Figure 1:**
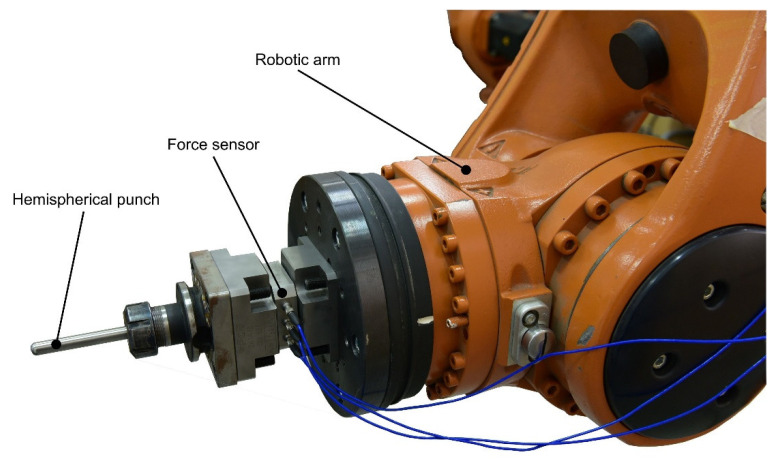
The PCB 261A13 force sensor mounted on the robotic arm of Kuka Kr210-2.

All research strictly related to the incremental forming process of polymeric materials was carried out using the Kuka Kr210-2 robot (Figure 1) (Augsburg, Germany). In fact, three options were available to achieve the kinematics required to produce parts by incremental forming: the use of a numerically controlled machine, of dedicated equipment for incremental forming or of an industrial robot. In the absence of dedicated equipment, the only options left were a CNC machine or a robot. Both options provided their own advantages, as well as disadvantages.

The CNC machine has higher rigidity but does not produce high forces in the x and y directions as it is not designed for plastic deformation processes. Moreover, one of the aims of the study is to measure the strains during the deformation process, which would not be possible due to the fact that the optical cameras of the measuring system cannot be positioned under the CNC machine table. The main disadvantage of robots is their lack of rigidity, but the vertical positioning of the semi-finished product would allow for the positioning of optical cameras. This problem can be significant in the case of hard materials with high tensile strength. However, lack of stiffness and hence precision is not an impediment in the case of polymeric materials, as they have a lower tensile strength and consequently develop lower deformation forces.

The Kuka KR210-2 robot was therefore chosen, a 6-axis robot with a maximum payload of 2100 N and a positioning repeatability of ±0.06 mm. The total weight of the robot is 1445 kg, and the total work volume is 97 m^3^. The robot is equipped with a KR C2 controller, 2005 edition.

The kinematics of the robot, required for the incremental forming processing of polymeric materials, are based on the desired model of the part, designed in Catia and exported to SprutCam, a program dedicated to generating by numerically controlled programs. A single type of trajectory was developed, a spiral trajectory for the production of truncated cone type parts. The deformation speed was of 400 mm/min.

The blank clamping system consists of a vertical frame and a clamping plate between which the semi-finished product is rigidly fixed. The free area of the blank is of 200 mm. The workpieces are in the form of square plates, 3 mm thick with sides of 250 mm.

To measure the forces in the incremental forming process of polymeric materials, a measuring system consisting of the PCB261A13 sensor (PCB piezotronics, Depew, NY, USA), the CMD600 signal amplifier (HBM, Darmstadt, Germany) and the Quantum X MX840B acquisition system (HBM, Darmstadt, Germany) was used. The force sensor allows force acquisition, and the amplifier increases the amplitude of the signal up to the range of voltages required for acquisition with the Quantum X system.

The PCB 261A13 force sensor produced by Piezotronics is specially designed to measure dynamic and quasi-static forces in three perpendicular directions simultaneously [54]. The sensor uses a set of quartz crystals arranged in a predefined pattern to optimize the measurement accuracy. The sensor features a classic three-directional piezoelectric sensor, clamped between two high-precision plates. Measurements in the z-axis direction are proportional to the compression, tensile or impact forces, while those in the x- and y-axis directions are proportional to the shear forces applied to the sensor.

In order to bring the signal acquired from the force sensor into the ±10 V voltage range, the CMD600 amplifier from HBM was used [55]. Three such amplifiers were used for the experimental research, one for each channel (for each of the Fz, Fx and Fy forces). This amplifier model is dedicated to piezoelectric sensors and allows the use of sensors with a detection range of 50 pC to 600,000 pC.

The Quantum X MX840B acquisition system is an acquisition system developed by HBM and features 8 individually configurable and galvanically isolated measurement channels [56]. The system allows individual sample rates of up to 40 kS/s per channel and has a built-in active low pass filter (Bessel, Butterworth). It is equipped with a 24-bit Analog to Digital converter on each channel. The Catman software, provided with the acquisition system, was used for the acquisition of the electrical signal.

In order to evaluate the formability during the single point incremental forming process, the uniaxial tensile test was used for the three materials, namely polyamide, polyethylene and polytetrafluoroethylene. They were uniaxially stressed with constant speed until fracture occurred, in order to primarily determine the maximum tensile stress and the tensile strain at maximum tensile stress. Different behaviors were observed in the tensile tests of the three materials, both in terms of the values obtained and in terms of the conventional stress–strain curves.

## 3. Results and Discussion

### 3.1. Tensile Tests

Five sets of specimens were made for each of the three materials (polyamide, polyethylene and polytetrafluoroethylene), which were then subjected to tensile tests. The uniaxial tensile test was conducted in accordance with ISO 527-1:2019 which covers the general principles for testing plastic materials [57]. In addition, the shape of the specimen and the testing speed were chosen in accordance with ISO 527-3:2018 which defines this data for polymer sheets and films [58]. After the tensile tests were performed, the data obtained was statistically processed using the Minitab software. The data was therefore studied under two aspects, namely the elimination of outliers (outliers test) and the normal data distribution (normality test). Two tests were actually applied: the Anderson–Darling test to check for the normal distribution of the data and the Grubbs test to remove outliers. The mean value and the standard deviation (St. dev) were initially calculated.

The Anderson–Darling test is a statistical test used to determine whether or not a data set follows a particular probability distribution, for example, the normal distribution, considered in this thesis. The test involves calculating the Anderson–Darling indicator (AD value).

The two hypotheses for the Anderson–Darling test for normal distribution are the following:

**Hypothesis** **0** **(H0).** *The data follow the normal distribution (null hypothesis).*

**Hypothesis** **1** **(H1).** *The data do not follow the normal distribution (alternative hypothesis).*

The null hypothesis assumes that the data are normally distributed, while the alternative hypothesis assumes that the data are non normal. In most cases, a *p*-value is determined for the Anderson–Darling test (*p*-value for AD test), which can be used to determine whether the test is significant or not. For low *p*-values (e.g., ≤0.05), the data is concluded to not follow the normal distribution. The *p*-value depends on the significance level chosen, although the vast majority of cases use the value of 0.05, corresponding to a probability of 95%.

The Grubbs test is a test used to identify and eliminate outliers. It is employed to detect outliers in a data set by testing for one outlier at a time. Any detected outliers are deleted from the data set and the test is repeated until no more outliers are detected.

The basic hypothesis underlying the Grubbs test is that, outliers aside, the data are normally distributed. The null hypothesis is that there are no outliers in the data set. It is worth mentioning that the Grubbs method only works when testing the most extreme value in the sample. A *p*-value (*p*-value for Grubbs test) is calculated for the Grubbs test as well. Normally, an indicator of the Grubbs test (G value) is calculated for all values, and the smallest *p*-value corresponding to the largest G-value is estimated. This *p*-value represents the chance of observing a point so far away from the others if the data were all sampled from a Gaussian distribution.

**Figure 2:**
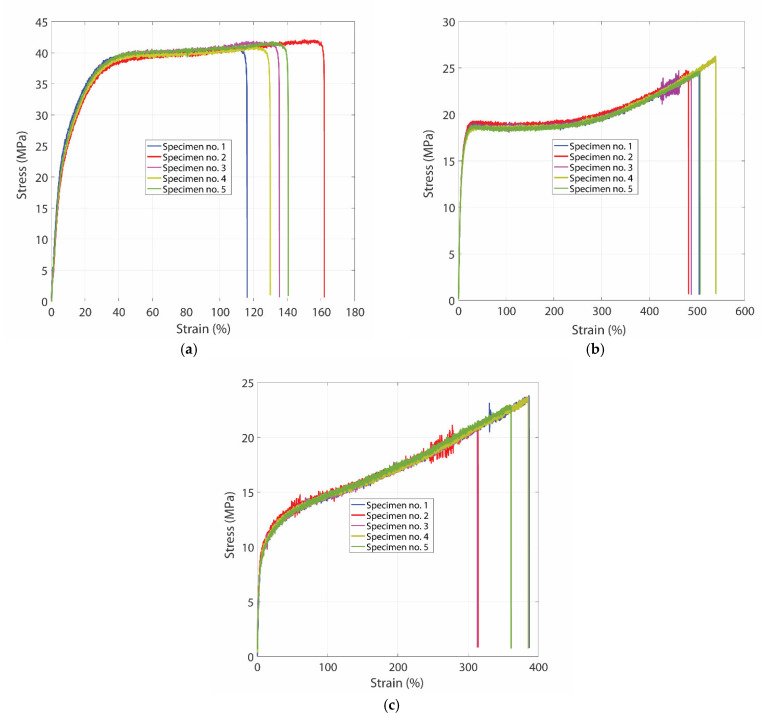
Stress–strain curves for polyamide (**a**), polyethylene (**b**) and polytetrafluorethylene (**c**).

Based on the previous considerations, the measured values and statistical processing of the two tests (Anderson–Darling and Grubbs for the three types of materials) are presented in Table 1, Table 2 and Table 3. The conventional characteristic stress–strain curves for all three tested materials are shown in Figure 2a–c. Due to the particularities of plastic flow in the cases of polyamide and polytetrafluoroethylene, it was not possible to determine the Yield stress, which is entirely accurate, as the observation of the conventional tensile graphs shows that these materials have practically no “Yield stress”, but a flow area that is not exactly defined.

The analysis of the results and statistical processing of the tensile tests of the four types of polymeric materials found no outliers for any of the measurements and a normal distribution of results for all obtained data. This is evidenced by the fact that the *p*-values are significantly greater than 0.05 in all cases.

The material with the lowest value of the elastic modulus is polytetrafluoroethylene (with an average value of 478.14 MPa), which indicates the fact that polytetrafluoroethylene has a much more noticeable elastic behavior. The Yield stress, which is only significant for polyethylene, averages 12.63 MPa, about 50% of the maximum value of the tensile stress in the material. The material with the highest value of the maximum tensile stress is polyamide with 41.54 MPa, followed by polyethylene and polytetrafluoroethylene with 24.73 MPa and 22.83 MPa, respectively. The material with the highest elongation, estimated on the basis of the tensile strain at maximum stress, is polyethylene with 496.7%, followed by polytetrafluoroethylene with 344.23% and polyamide with 126.53%.

Based on the observations presented above, the research on the behavior during SPIF was continued for all three polymeric materials previously discussed.

### 3.2. Force Determination during Incremental Forming of Polymeric Materials

One of the most important output parameters for the incremental forming process is the force developed in the process. The force during the incremental forming process, in contrast to conventional forming processes (e.g., deep drawing), consists of three components that vary significantly throughout the process. Therefore, the force measurement in the incremental forming of polymeric materials made necessary the use of the force sensor together with the conditioning module and the data acquisition system.

Based on the literature review, the main factors influencing the forces in the incremental forming process of polymeric materials were selected. These factors are presented in Table 4.

Of the seven input factors presented in Table 4, only the first four, namely the material type, the punch diameter, the step on vertical direction and the wall angle were selected for analysis for a number of reasons: two or more identical initial thicknesses were not available for all materials, the use of an industrial robot did not allow for a rotational movement of the punch and the value used for the punch feed was the maximum value, in order to reduce the processing time.

**Figure 3:**
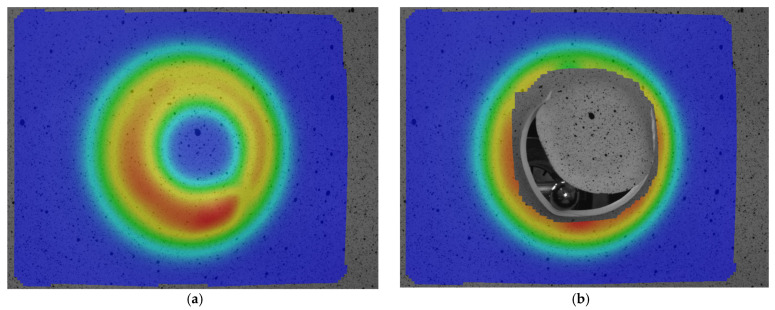
The images of the part manufactured by polytetrafluorethylene with wall angle α = 50°, punch diameter Dp = 8 mm and step s = 0.75 mm taken before (**a**) and immediately after (**b**) failure.

Previous to designing the experiments, given that the first four parameters presented in Table 4 were to be considered, several preliminary experiments were carried out, attempting to identify the limits of the process in terms of formability. For this reason, three sets of experiments were performed for all three types of materials. The first set had the following configuration: wall angle α = 60°, punch diameter Dp = 10 mm and step s = 0.75 mm. The second set had the following input parameters: wall angle α = 55°, punch diameter Dp = 6 mm and step s = 0.75 mm, while the third one had: wall angle α = 50°, punch diameter Dp = 8 mm and step s = 0.75 mm. In fact, these experiments were conducted to observe whether, at different angles of the part and different diameters of the punch, all materials allow deformation without breaking. The step was set constant at an average value of 0.75 mm. These preliminary tests proved useful as they found that polytetrafluoroethylene broke in all 3 variations of the experiment. Figure 3a,b, show images of the part taken before and immediately after failure for the third set of experiments (wall angle α = 50°, punch diameter Dp = 8 mm and step s = 0.75 mm).

A significant decrease in formability was observed for polytetrafluoroethylene, leading to the conclusion that this material is not suitable for incremental forming at room temperature. Possible future research could address the hot deformation of this material. As a result, it was decided to eliminate polytetrafluoroethylene from the experimental investigations and only keep polyamide and polyethylene.

#### 3.2.1. Analysis of the Signal-to-Noise Ratio for the Forces during the Incremental Forming of Polymeric Materials

For the design of experiments, there are software packages that automatically perform the experimental plan using: a general full-factorial experiment, a fractional experiment, a surface response methodology or the Taguchi methodology. The Taguchi method is derived from the factorial methodology and throughout time it has proven its efficiency over other methods in the design of experiments. For the study presented below the Taguchi design of experiment was chosen because of its most significant advantage over other methods, i.e., obtaining robust results with a small number of experiments.

Before outlining the experimental investigations using the Taguchi orthogonal arrays, it is worth mentioning the fact that in the general case of forming processes (not only in the particular case of incremental forming), the minimization of forces is always desired. This is because, in the case of forming processes, the machine is chosen according to the required force. All the more so in the present case, where an industrial robot which does not produce a force as great as that of a press or of a CNC machine, nor has a very high rigidity was used in the experiments, it is important to be able to reduce the forces in the process. 

Therefore, the aim of the experimental investigation is to decrease the maximum force values found in the peaks of the variation graphs. The design of experiments based on the Taguchi method is used to analyze the effect of the input processing parameters on the forces during the incremental forming processing of polymeric materials. Four parameters were selected from the input parameters presented in Table 4, of which two parameters are related to the part to be produced and the other two are technological parameters, related to the operating regime used. The parameters concerning the part are the material of the part (MAT) and the angle of the part produced (α), whereas the technological parameters are the diameter of the punch (Dp) and the step on vertical direction (s). 

The material of the part introduces two levels of variation, with polyamide and polyethylene having been selected as materials, as mentioned previously, while the other parameters are defined in three levels of variation, namely 50°, 55° and 60° for the angle of the part, 6 mm, 8 mm and 10 mm for the diameter of the punch and 0.5 mm, 0.75 mm and 1 mm for the step on vertical direction. The values chosen for the step may raise an issue. In the case of incremental forming of metallic materials, the step chosen has smaller values (0.1 mm, 0.25 mm) since a larger step would lead to “waves” on the inner surface of the part. Given the fact that polymeric materials have a very strong springback ability and that a small step size would lead to particularly long processing times, the step size values presented above were chosen in order to increase the productivity of the process. 

To study the contribution of each input parameter on the values of the force during the incremental forming of polymeric materials, the Taguchi orthogonal array L18(2^1^ × 3^3^) with mixed levels (one parameter has two levels of variation and three parameters have three levels of variation) is selected. Table 5 contains the design of experiments, with the factors and levels of variation, as well as their codifications. To reduce the margin of error, all eighteen experiments were repeated twice. The forces in the process were measured along all three directions of the coordinate axes.

Depending on the purpose of the experiments, different signal-to-noise (S/N) ratios can be selected as follows: “smaller is better”, “larger is better” and “nominal is better”. For this study, as already mentioned, the “smaller is better” option was chosen for all forces present in the process.

The numerical results of the measurements, namely the mean value, standard deviation, S/N ratio and variation coefficient for all three types of forces are presented in Table 6, Table 7 and Table 8.

The force variation graphs for the F2, F9, F11 and F18 cases, two for polyamide (F2 and F9) and two for polyethylene (F11 and F18), obtained from the design using the Taguchi orthogonal array for the three directions of the coordinate axis system, are shown in Figure 4, Figure 5, Figure 6, Figure 7, Figure 8, Figure 9, Figure 10, Figure 11, Figure 12, Figure 13, Figure 14 and Figure 15.

**Figure 4:**
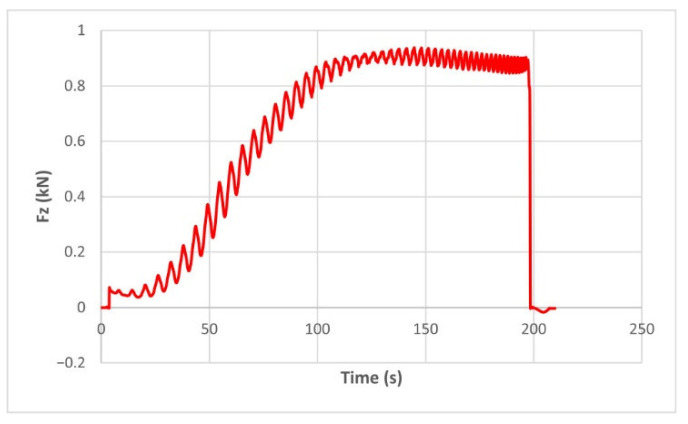
The Fz force variation for the parts produced from PA, with Dp = 8 mm, s = 0.75 and α = 50° (case F2).

**Figure 5:**
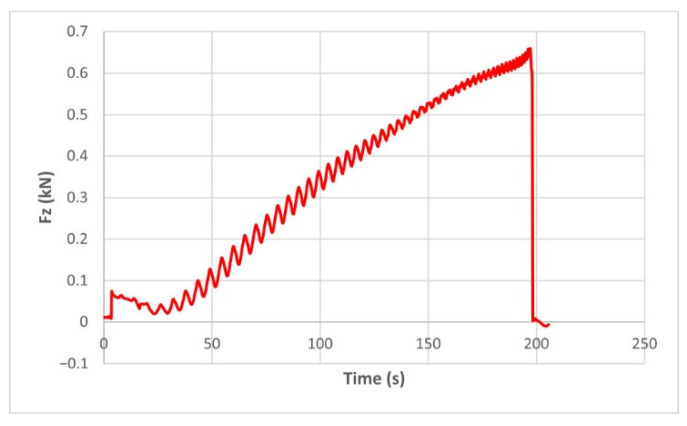
The Fz force variation for the parts produced from PE, with Dp = 8 mm, s = 0.75 and α = 50° (case F11).

The force in the Oz direction (Fz) increases for both materials, with local maxima and minima from the first contact of the punch with the blank, as can be seen in Figure 4, Figure 5, Figure 10 and Figure 11. The local maxima occur in the areas where the punch penetrates the material by one step, while the minima occur in the moment prior to this moment. However, a slightly different behavior is observed for the two materials. In the case of polyamide, the force increases with a steeper slope, reaching a maximum value somewhere towards the middle of the working stroke and then remains relatively constant or even decreases slightly when using punches with smaller diameters. This is most noticeable when the punch has a 6 mm diameter (cases F1, F4 and F7). When it comes to polyethylene, the force increases with a less steep slope, slowly reaching its maximum value somewhere at the end of the working stroke, where it remains relatively constant. When using a 10 mm diameter punch (cases F12, F15 and F18), the force increases continuously all the way to the end.

**Figure 6:**
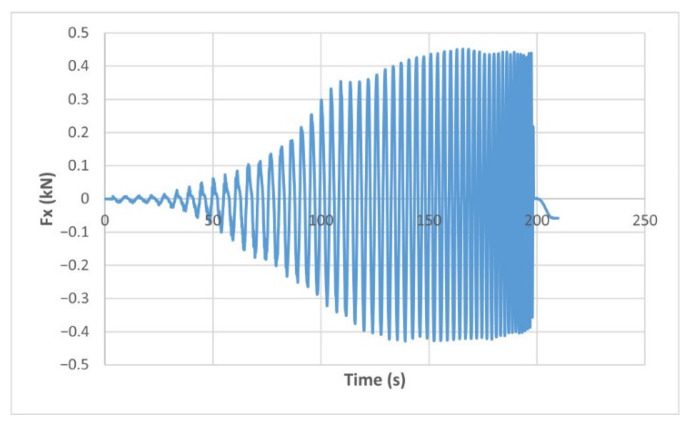
The Fx force variation for the parts produced from PA, with Dp = 8 mm, s = 0.75 and α = 50° (case F2).

**Figure 7:**
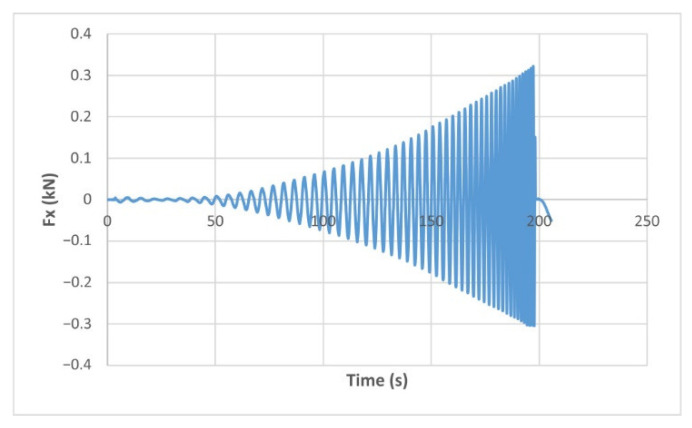
The Fx force variation for the parts produced from PE, with Dp = 8 mm, s = 0.75 and α = 50° (case F11).

**Figure 8:**
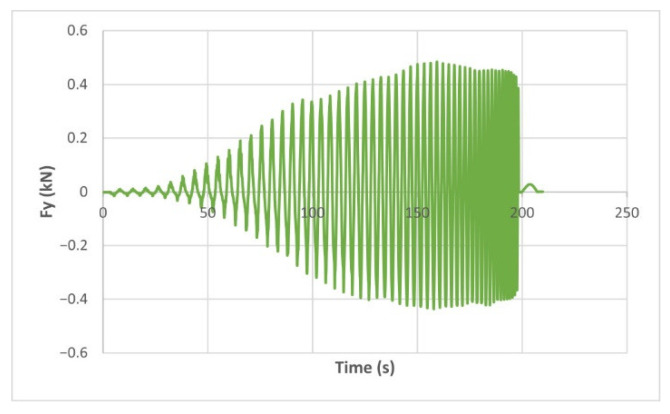
The Fy force variation for the parts produced from PA, with Dp = 8 mm, s = 0.75 and α = 50° (case F2).

**Figure 9:**
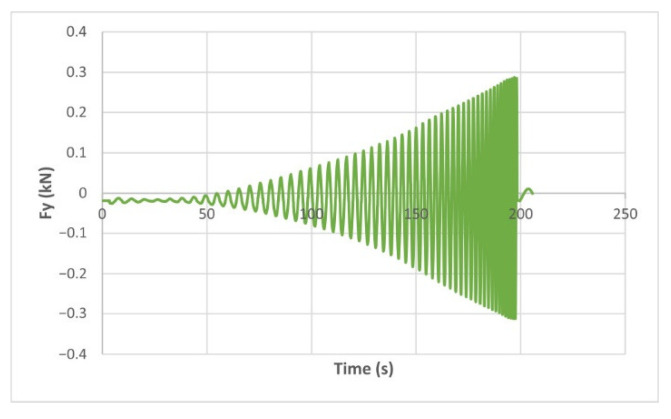
The Fy force variation for the parts produced from PE, with Dp = 8 mm, s = 0.75 and α = 50° (case F11).

**Figure 10:**
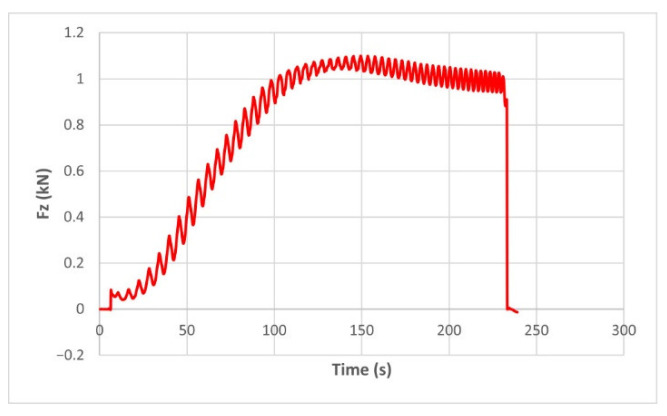
The Fz force variation for the parts produced from PA, with Dp = 10 mm, s = 0.75 and α = 60° (case F9).

**Figure 11:**
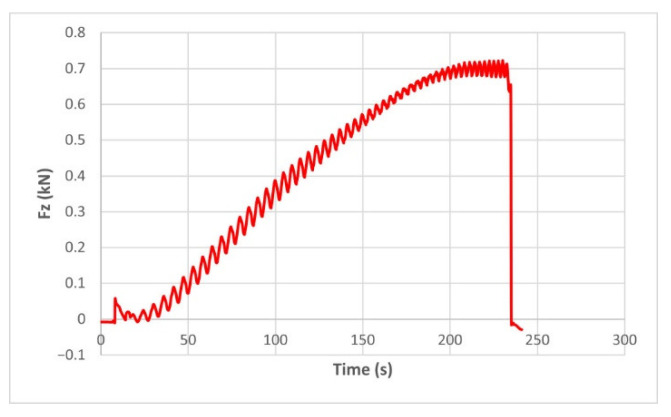
The Fz force variation for the parts produced from PE, with Dp = 10 mm, s = 0.75 and α = 60° (case F18).

**Figure 12:**
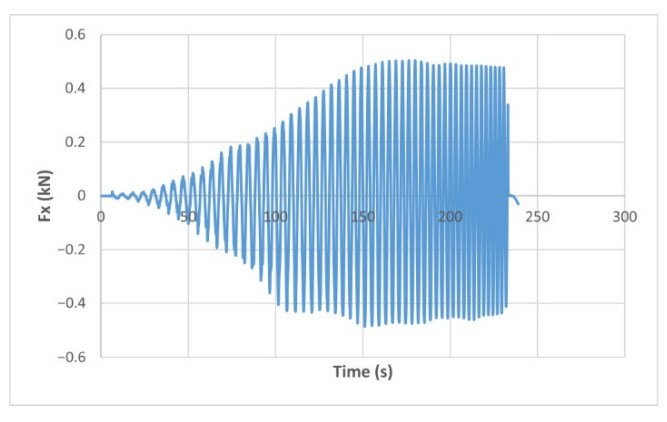
The Fx force variation for the parts produced from PA, with Dp = 10 mm, s = 0.75 and α = 60° (case F9).

**Figure 13:**
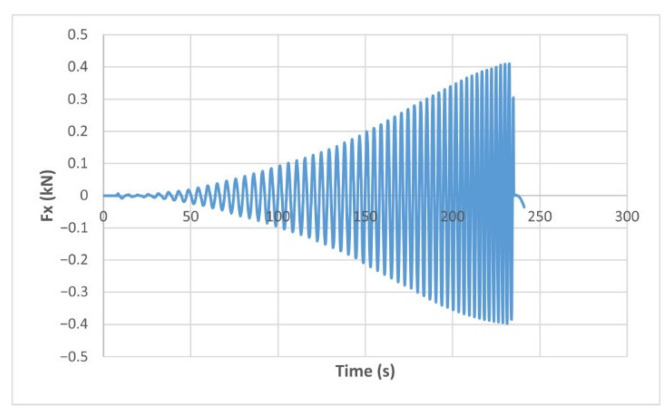
The Fx force variation for the parts produced from PE, with Dp = 10 mm, s = 0.75 and α = 60° (case F18).

**Figure 14:**
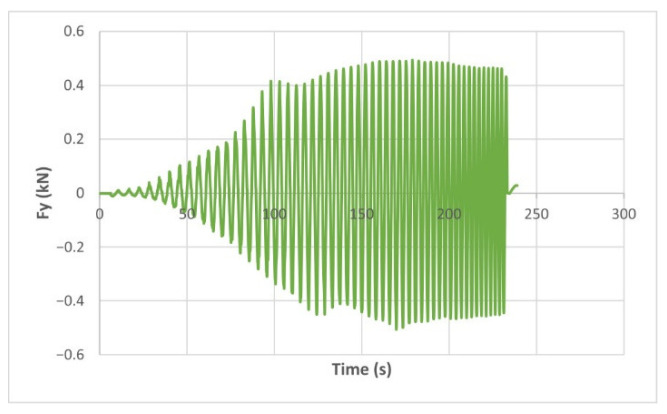
The Fy force variation for the parts produced from PA, with Dp = 10 mm, s = 0.75 and α = 60° (case F9).

**Figure 15:**
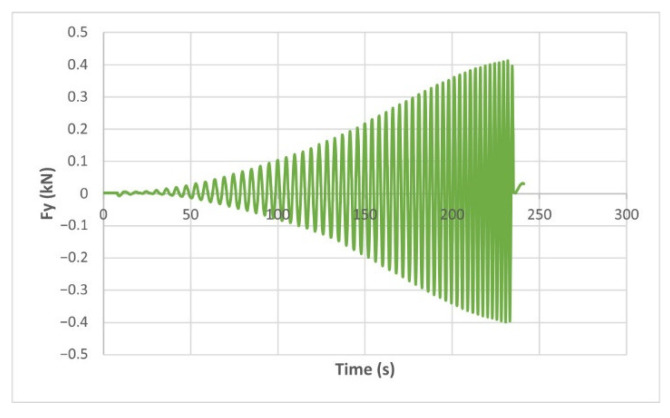
The Fy force variation for the parts produced from PE, with Dp = 10 mm, s = 0.75 and α = 60° (case F18).

**Figure 16:**
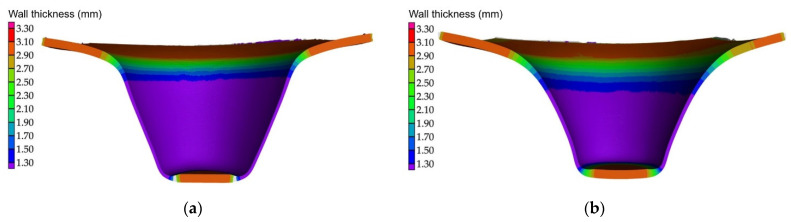
The CT scan of the parts obtained for cases F9—polyamide (**a**) and F18—polyethylene (**b**).

Forces in the Ox and Oy directions (Fx) and (Fy) have a somewhat similar variation from each other, but different to that of the Fz force. Basically, the two forces increase and decrease around zero, reaching local maxima and minima with every circle the punch travels in the horizontal plane, as can be seen in Figure 6, Figure 7, Figure 8, Figure 9, Figure 12, Figure 13, Figure 14 and Figure 15. The behavior of the two analyzed materials is slightly different here also. For polyamide, like for the Fz force, the local maxima of the Fx and Fy forces also increase to about half the stroke value and then stabilize until the end of the process. In the case of polyethylene, the stagnation towards the end of the working stroke is not so evident, with the values of the local maxima rising continuously until near the end. After the manufacturing process, the incrementally formed parts were CT scanned. The results of the scanning process for cases F9 and F18 are presented in Figure 16.

As can be seen in Figure 16, using the same trajectory for both materials, different geometries are obtained. The polyamide part results with a geometry similar to a frustum of cone (as it is desired), while the polyethylene part results with a concave geometry. For this reason, even though the parts have the same depth, the diameter of the section is smaller for polyethylene than for polyamide. It is for this reason that the variation of the forces follows a different pattern for the two polymeric materials.

**Figure 17:**
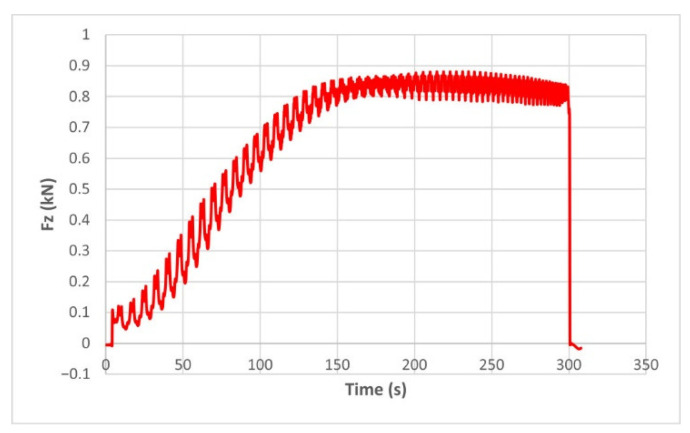
The Fz force variation for the frustum of pyramid parts produced from PA, with Dp = 8 mm, s = 0.75 and α = 50°.

**Figure 18:**
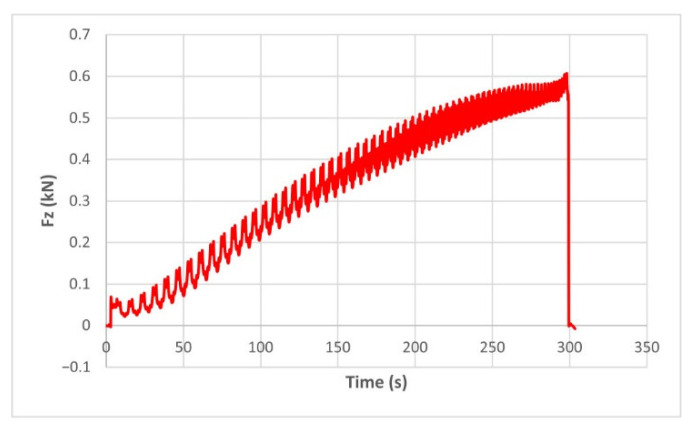
The Fz force variation for the frustum of pyramid parts produced from PE, with Dp = 8 mm, s = 0.75 and α = 50°.

**Figure 19:**
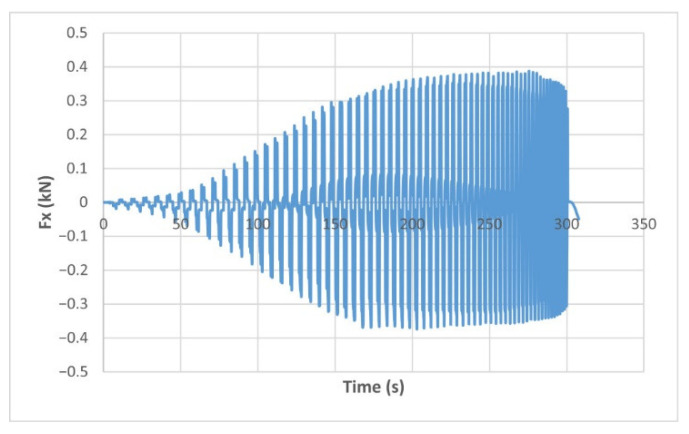
The Fx force variation for the frustum of pyramid parts produced from PA, with Dp = 8 mm, s = 0.75 and α = 50°.

**Figure 20:**
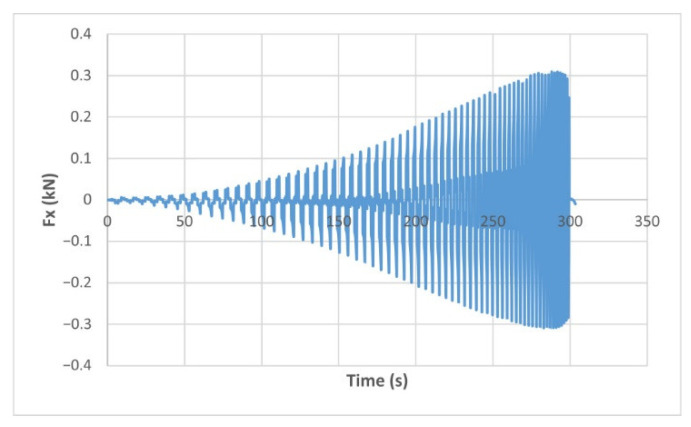
The Fx force variation for the frustum of pyramid parts produced from PE, with Dp = 8 mm, s = 0.75 and α = 50°.

**Figure 21:**
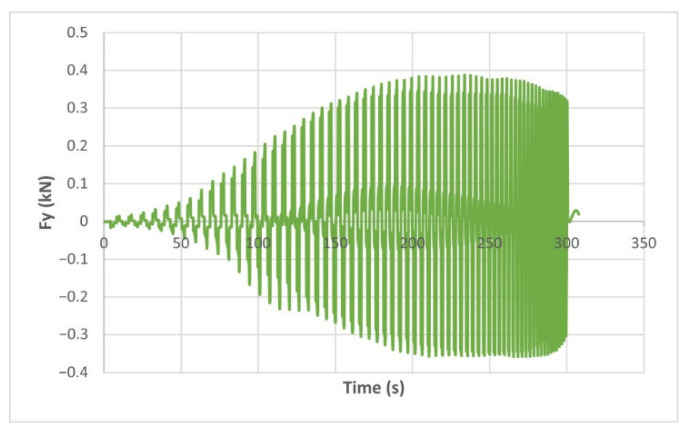
The Fy force variation for the frustum of pyramid parts produced from PA, with Dp = 8 mm, s = 0.75 and α = 50°.

**Figure 22:**
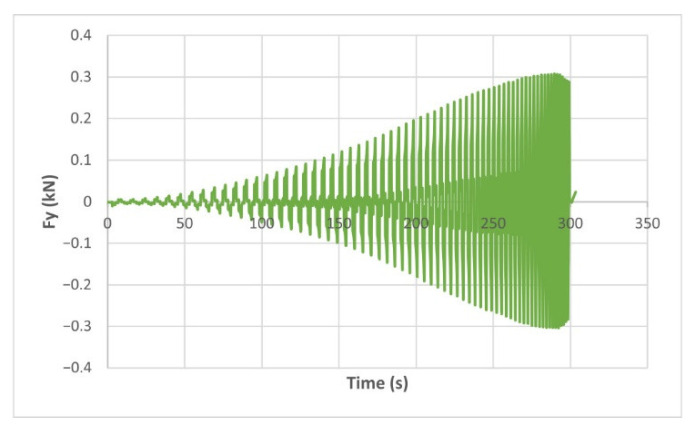
The Fy force variation for the frustum of pyramid parts produced from PE, with Dp = 8 mm, s = 0.75 and α = 50°.

The maximum values of the forces are always higher in the case of polyamide than in the case of polyethylene, for both the Fz force and the Fx and Fy forces. Thus, the maximum value of the Fz force occurs in case F3 (1.2173 kN), while those of the Fx and Fy forces are also obtained in case F3, with 0.5842 kN for Fx and 0.5908 kN for Fy, respectively. The minimum values of the forces occur in case F10—polyethylene (Fz = 0.4371 kN, Fx = 0.2066 kN and Fy = 0.2061 kN, respectively). Two parts made of polyamide and polyethylene with a frustum of pyramid type trajectory were also made, with the following input parameters: Dp = 8 mm, s = 0.75 mm and α = 50°, corresponding to cases F2 and F11 in Table 8. The variation graphs of the six forces (three for each material) are shown in Figure 17, Figure 18, Figure 19, Figure 20, Figure 21 and Figure 22.

The analysis of the graphs for the frustum of pyramid type parts leads to the same conclusions as those for the frustum of cone type parts, i.e., in the case of polyamide the force reaches its maximum value at about half of the working stroke, after which it stays constant or even decreases, as shown in Figure 17, while in the case of polyethylene it increases constantly until close to the end of the working stroke, as shown in Figure 18. 

The maximum Fz force values obtained for the frustum of pyramid type parts are lower for both polyamide (0.8810 kN) and polyethylene (0.6083 kN) than those obtained for the frustum of cone type parts (0.9365 kN—polyamide, 0.6576 kN—polyethylene). The maximum Fx force values obtained for the frustum of pyramid type parts are also lower for both polyamide (0.3876 kN), as shown in Figure 19, and polyethylene (0.3087 kN), as shown in Figure 20, compared to those obtained for the frustum of cone type parts (0.4509 kN—polyamide, presented in Figure 12, 0.3252 kN—polyethylene, presented in Figure 13). The same applies in the case of the Fy force, where the values for polyamide (0.3879 kN), presented in Figure 21, and polyethylene (0.3076 kN), presented in Figure 22, are lower than those obtained for the frustum of cone type parts (0.4553 kN—polyamide, as shown in Figure 14, 0.3270 kN—polyethylene, as shown in Figure 15).

The mean response values of the signal-to-noise ratios (S/N) for the Fz force (with the “smaller is better” condition) are shown in Table 9.

Table 9 provides the mean response values of the S/N ratios, which analyze the effect of the influencing factors (MAT, α, Dp and p) on the force in the z-direction (Fz). It shows the optimal levels (based on the S/N ratios) for which the control factors result in the lowest value of Fz. These are: level 2 for MAT (S/N = 4.271), level 3 for α (S/N = 2.602), level 1 for Dp (S/N = 4.314) and level 1 for s (S/N = 3.498). Delta and rank determine which of these four factors has the greatest impact on the response. Delta measures the magnitude of impact by subtracting the lowest mean response value of a factor from its highest value. Rank assigns an order number to the factors, from the highest to the lowest impact, based on their delta values. For Fz, the material has the highest impact (delta: S/N = 3.538 and rank = 1), but the punch diameter also has a very similar impact (delta: S/N = 3.535 and rank = 2), while α has the lowest impact (delta: S/N = 0.327 and rank = 4).

The mean response values of the signal-to-noise ratios (S/N) for the Fx force (with the “smaller is better” condition) are shown in Table 10.

Similar to the case presented for the Fz force, Table 10 indicates the optimal levels of the control factors for which the lowest value of Fx is obtained. Thus, the optimal values are obtained for MAT at level 2 (S/N = 9.665), α at level 1 (S/N = 8.605), Dp at level 1 (S/N = 9.741) and s at level 1 (S/N = 9.687). The material has the highest impact for Fx as well (delta: S/N = 3.099 and rank = 1), with the punch diameter again having a similar impact (delta: S/N = 3.085 and rank = 2), while α has the lowest impact (delta: S/N = 0.706 and rank = 4).

The mean response values of the signal-to-noise ratios (S/N) for the Fy force (with the “smaller is better” condition) are shown in Table 11.

An analysis of Table 11 leads to the optimal values resulting in the lowest value of Fy: MAT at level 2 (S/N = 9.633), α at level 1 (S/N = 8.518), Dp at level 1 (S/N = 9.681) and s at level 1 (S/N = 9.588). For Fy, as for Fx, the material has the highest impact (delta: S/N = 3.058 and rank = 1), followed by the punch diameter (delta: S/N = 3.013 and rank = 2), with α having the lowest impact (delta: S/N = 0.649 and rank = 4).

**Figure 23:**
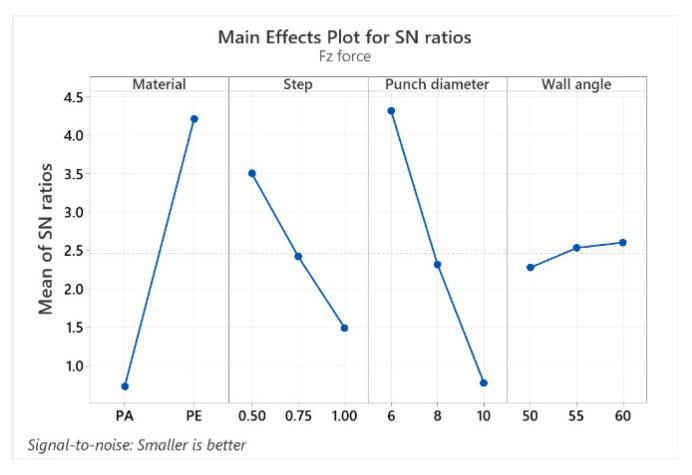
The main effects plot for S/N ratios for Fz force.

**Figure 24:**
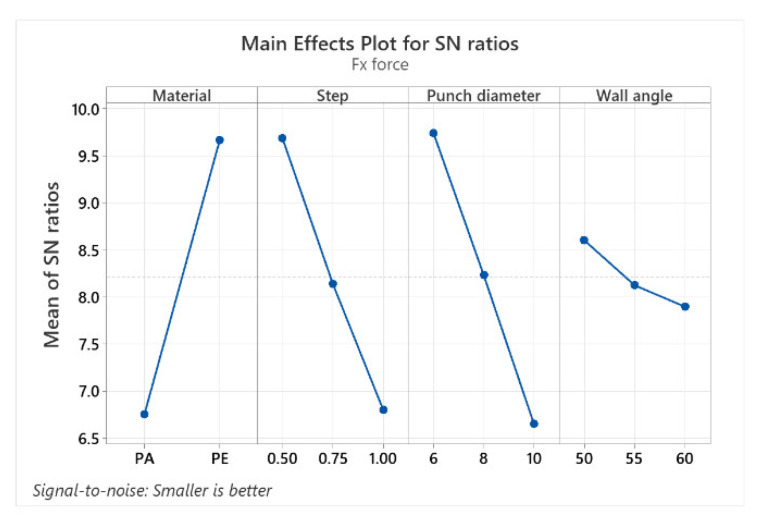
The main effects plot for S/N ratios for Fx force.

**Figure 25:**
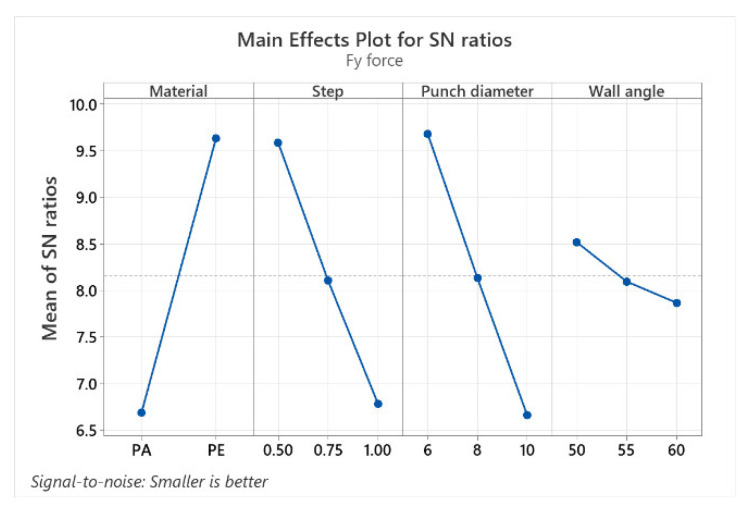
The main effects plot for S/N ratios for Fy force.

The graphical representation (the graph showing the main effects for the signal-to-noise (S/N) ratios) of the factor levels in Table 9, Table 10 and Table 11 for the three forces (Fz, Fx and Fy) is shown in Figure 23, Figure 24 and Figure 25.

The graphs of the main effects show the optimal values of the control factors that lead to the lowest values of the forces. The main effect is also called the difference between means, caused by the different levels within a factor. If the graph line is horizontal, then there is no main effect for the levels of a factor, but a line with a greater slope or difference between vertical points will have a greater main effect.

For all three categories of forces, the main effect graphs for the material (MAT) and for the punch diameter (Dp) display graph lines with larger slopes, therefore having the largest main effect. The main goal of the experimental investigations is to find the control factor levels that minimize the impact of the noise factors on the responses. The optimal parameters for minimizing the forces can be easily determined from these graphs.

The best level for each control factor was found based on the highest S/N ratio in the levels of these input parameters (control factors). The range of the control factors throughout the experiments was mentioned earlier in this chapter. The analysis of Table 9, Table 10 and Table 11, as well as of the graphs showing the main effects, leads to the optimal values for which the Fz, Fx and Fy forces can be kept as low as possible. Thus, the optimal values for which the Fz force is minimal are the following: 6 mm for Dp, polyethylene for MAT, 0.5 mm for s and 60° for α. Minimum Fx and Fy forces are obtained with the following optimal values: Dp = 6 mm, MAT—polyethylene, s = 0.5 mm and α = 50°.

#### 3.2.2. Analysis of Variance for the Forces during the Incremental Forming of Polymeric Materials

For further robustness of the results obtained by the Taguchi method based on the S/N ratios, an ANOVA statistical model is used. Unlike the Taguchi method, it calculates the contribution of each factor involved. In addition, ANOVA analysis also allows the assessment of interactions between different factors. For the present case, the interactions between the material and the technological factors, namely the diameter of the punch and the step, were chosen. The significance of the control factors in the ANOVA analysis is determined by comparing the F-values of each control factor. These tables were calculated with a 5% significance level and a 95% confidence level. If the *p*-value of the factor is greater than 0.05, then it will be considered a non-significant factor. Table 12 shows the ANOVA table for the Fz force.

An analysis of the *p*-values in Table 12 shows that a *p*-value greater than 0.05 occurs in the case of the part angle and of two interactions (material * punch diameter and material * step). These factors are therefore non-significant for the Fz force. Table 13 shows the ANOVA table for the Fx force.

The *p*-values in Table 13 indicate a *p*-value greater than 0.05 for only two interactions (material * punch diameter and material * step). Therefore, all other factors are significant for the Fx force. Table 14 presents the ANOVA table for the Fy force.

Considering the *p*-values in Table 14, only two interactions (material * punch diameter and material * step) have a *p*-value greater than 0.05, as in the case of the Fx force.

The percentage contribution in the ANOVA tables shows the magnitude of the influence of the parameters. The contribution of the material is 51.04%, of the punch diameter is 35.46%, of the step is 11.36% and of the angle is only 0.33% for the value of the Fz force. Similarly, for the value of the Fx force, these percentages are 40.3% for the material, 30.21% for the punch diameter, 26.47% for the step and 1.65% for the angle. As for the value of the Fy force, the percentage contributions are 41.94% for the material, 29.31% for the punch diameter, 25.32% for the step and 1.4% for the angle. The most significant parameter for all three responses is the blank material. The percentage of error was considerably low, 0.8% for Fz, 0.59% for Fx and 0.57% for Fy, respectively.

#### 3.2.3. Regression Analysis for the Forces during the Incremental Forming of Polymeric Materials

In order to obtain the mathematical relationships of the forces in the process for the input parameters, the two materials had to be separated and one relationship had to be obtained for each of them. Thus, this study resulted in finding relationships between the three response variables (forces Fz, Fx and Fy) and the three input variables (α, Dp and s). These are obtained by using the regression analysis. Three linear regression equations were modeled for each material. Several important parameters for evaluating regression models are R-sq (describes how much variation is present in the response), R-sq (adj) (is a modified R-sq that has been adjusted for the number of expressions), and R-sq (pred) (indicates how well the model predicts responses for new measurements). For the regression model to be of best performance, these values must be high. 

Following the regression analysis performed with Minitab, the data given in Table 15 was obtained for the Fz force in polyamide.

As can be seen from the *p*-values obtained, the coefficients of the angle α and of the constant in the regression equation are not significant, having values greater than 0.05. These coefficients are consequently excluded, and the analysis is repeated. The regression equation is then obtained by Equation (1):

(1) $$F_{z\_\mathrm{PA}}=0.07511\cdot D_{p}+0.4442\cdot s$$

An excellent predictability of the proposed mathematical model was achieved (R-sq (pred) = 99.89%). A regression analysis was then performed for the Fz force in the case of polyethylene, the results of which are shown in Table 16.

In this case also, the *p*-values indicate that the coefficients of the angle α and of the constant in the regression equation are not significant, since they are greater than 0.05. Therefore, again, these coefficients are eliminated, and the analysis is repeated, leading to the following regression equation, Equation (2):

(2) $$F_{z\_\mathrm{PE}}=0.06062\cdot D_{p}+0.2004\cdot s$$

Excellent predictability of the proposed mathematical model (R-sq (pred) = 99.09%) was also obtained in this case. Following the regression analysis performed with Minitab, the data presented in Table 17 for the Fx force in polyamide was obtained.

As can be seen from the *p*-values, all coefficients are significant, with values less than 0.05. This generates the following regression equation, Equation (3):

(3) $$F_{x\_\mathrm{PA}}=-0.1460+0.002402\cdot \alpha +0.03443\cdot D_{p}+0.2731\cdot s$$

The value of R-sq(pred) is 98.51%, which means that again a good predictability of the regression model was obtained. A regression analysis was then conducted for the Fx force in polyethylene, with the results presented in Table 18.

Similar to the Fx force in polyamide, it is observed that all coefficients are significant, with values less than 0.05. The following regression equation is then obtained by Equation (4):

(4) $$F_{x\_PE}=-0.1867+0.001637\cdot \alpha +0.03260\cdot D_{p}+0.2302\cdot s$$

In this case too, a good predictability of the proposed mathematical model is found (R-sq(pred) = 98.32%). The regression analysis performed with Minitab resulted in the data shown in Table 19 for the Fy force in polyamide.

The *p*-values show that all coefficients are significant. Therefore, the regression equation is as follows:

(5) $$F_{y\_\mathrm{PA}}=-0.0987+0.002193\cdot \alpha +0.032176\cdot D_{p}+0.2518\cdot s$$

The value obtained for R-sq(pred) is 99.15%, again indicating a good predictability of the regression model. The regression analysis for the Fy force in polyethylene leads to the results shown in Table 20.

Again, all coefficients are significant, having values less than 0.05. The regression equation obtained is:

(6) $$F_{y\_\mathrm{PE}}=-0.1891+0.001577\cdot \alpha +0.032989\cdot D_{p}+0.23512\cdot s$$

The value of R-sq(pred) is 99.07%, which means that this model also has a good predictability.

Both the Taguchi method and the analysis of variance led to the same conclusion: the most important influence on the maximum value of the force belongs to the material, followed by the diameter of the punch, the vertical step and finally the wall angle of the part for all three types of forces. In fact, the analysis of variance shows that the influence of the wall angle is 0.33% for the Fz force, 1.65% for the Fx force and 1.4% for the Fy force, and it can therefore be concluded that the angle of the part has almost no impact on the values of the three types of forces.

Knowledge of how the analyzed input parameters influence the force variation in the single point incremental forming process, and more importantly knowing the optimal values that lead to minimizing the values of these forces, is of great importance for the academic-scientific community of sciences and engineering. This is because it allows us to choose the optimal forming machine for the process, or in case the machine cannot be changed, it helps us to choose the optimal values of the input parameters in order to minimize the forces in the process, as well as the afferent energy consumption. As is well known, in material forming processes, unlike machining processes, the machine (press) is chosen on the basis of the force required in the process and not on the basis of the power available, which obviously leads to solving an important problem in this process. The most significant difference between the Taguchi method and the other methods consists of the manner in which noise is taken into account in an experiment. While the other statistical methods attempt to eliminate the noise, the Taguchi method uses a composite performance indicator, the signal to noise (S/N) ratio, which is used to identify the optimal combinations of input parameters that are most insensitive to noise.

The optimum values leading to minimum forces in the incremental forming of polymeric materials must be correlated with minimizing the defects of the polymeric parts. For this reason, this study needs further updating with a follow-up considering the influence of the input parameters (polymeric materials, wall angle, punch diameter and vertical step) with respect to the springback, wrinkles and twist defects.

## 4. Conclusions

The main objective of the paper was to determine the optimal values of the input parameters for which the forces during the single point incremental forming of polymeric materials are minimal. Based on the study on the signal-to-noise ratio of the Taguchi method and on the analysis of variance, it was found that the main forming force, perpendicular to the initial plane of the table, decreases the smaller the punch diameter, the smaller the value of the step and the larger the value of the wall angle of the part, while the forces on the directions of the initial plane of the table decrease the smaller the punch diameter, the smaller the value of the step and the smaller the value of the wall angle of the part. Another observation shows that the forces obtained in the case of polyethylene are always lower than in the case of polyamide when manufactured under the same forming conditions (the same punch diameter, step and wall angle).

## Figures and Tables

**Table 1:** Results of the tensile test for polyamide specimens.

Specimen Number	Young Modulus (MPa)	Yield Stress (MPa)	Maximum Tensile Stress (MPa)	Tensile Strain at Maximum Tensile Stress (%)
1.	1820.545	-	40.989	110.833
2.	1869.364	-	42.112	150.000
3.	1839.354	-	41.824	119.999
4.	1710.215	-	41.013	120.500
5.	1896.895	-	41.781	131.333
Mean	1827.274	-	41.544	126.533
St. dev	71.611	-	0.512	14.994
AD value	0.335		0.436	0.307
*p*-value for AD test	0.334	-	0.166	0.401
G value	1.630	-	1.110	1.570
*p*-value for Grubbs test	0.150	-	1.000	0.261

**Table 2:** Results of the tensile test for polyethylene specimens.

Specimen Number	Young Modulus (MPa)	Yield Stress (MPa)	Maximum Tensile Stress (MPa)	Tensile Strain at Maximum Tensile Stress (%)
1.	1044.393	12.699	24.339	502.167
2.	1068.163	12.692	24.735	476.667
3.	914.544	12.565	24.683	461.667
4.	961.303	12.613	25.241	538.833
5.	1035.568	12.574	24.651	504.167
Mean	1004.794	12.629	24.730	496.700
St. dev	64.357	0.064	0.325	29.536
AD value	0.330	0.389	0.373	0.219
*p*-value for AD test	0.345	0.231	0.258	0.673
G value	1.400	1.110	1.570	1.430
*p*-value for Grubbs test	0.583	1.000	0.247	0.530

**Table 3:** Results of the tensile test for polytetrafluoroethylene specimens.

Specimen Number	Young Modulus (MPa)	Yield Stress (MPa)	Maximum Tensile Stress (MPa)	Tensile Strain at Maximum Tensile Stress (%)
1.	472.717	-	23.796	386.499
2.	535.948	-	22.532	277.833
3.	471.926	-	21.166	312.500
4.	485.780	-	23.671	385.500
5.	424.333	-	22.959	358.833
Mean	478.141	-	22.825	344.233
St. dev	39.885	-	1.062	47.745
AD value	0.299		0.294	0.328
*p*-value for AD test	0.425	-	0.439	0.349
G value	1.450	-	1.560	1.390
*p*-value for Grubbs test	0.482	-	0.267	0.609

**Table 4:** The main influence factors and their level of variation.

Input Factors					Output Factors
Code	Type	Symbol	Unit	Variation Domain	y_1_, y_2_, y_3_
x_1_	Polymeric material	MAT	-	PA, PE, PTFE	Fz, Fx, Fy (kN)
x_2_	Step	s	(mm)	0.5–1	Fz, Fx, Fy (kN)
x_3_	Punch diameter	Dp	(mm)	6–10	Fz, Fx, Fy (kN)
x_4_	Wall angle	α	(°)	50–60	Fz, Fx, Fy (kN)
x_5_	Initial thickness	g	(mm)	0.5–5	Fz, Fx, Fy (kN)
x_6_	Punch feed	v_s_	(mm/min)	100–600	Fz, Fx, Fy (kN)
x_7_	Punch angular speed	ω	(rot/min)	50–180	Fz, Fx, Fy (kN)

**Table 5:** The design of experiments using Taguchi orthogonal array L18 (2^1^ × 3^3^).

Case	Mat. Code	α Code	Dp Code	s Code	Mat.	α (°)	Dp (mm)	s (mm)
F1	−1	−1	−1	−1	PA	50	6	0.50
F2	−1	−1	0	0	PA	50	8	0.75
F3	−1	−1	1	1	PA	50	10	1.00
F4	−1	0	−1	0	PA	55	6	0.75
F5	−1	0	0	1	PA	55	8	1.00
F6	−1	0	1	−1	PA	55	10	0.50
F7	−1	1	−1	1	PA	60	6	1.00
F8	−1	1	0	−1	PA	60	8	0.50
F9	−1	1	1	0	PA	60	10	0.75
F10	1	−1	−1	−1	PE	50	6	0.50
F11	1	−1	0	0	PE	50	8	0.75
F12	1	−1	1	1	PE	50	10	1.00
F13	1	0	−1	0	PE	55	6	0.75
F14	1	0	0	1	PE	55	8	1.00
F15	1	0	1	−1	PE	55	10	0.50
F16	1	1	−1	1	PE	60	6	1.00
F17	1	1	0	−1	PE	60	8	0.50
F18	1	1	1	0	PE	60	10	0.75

**Table 6:** The results and the statistical processing for the Fz forces.

Cod	Mat.	α (°)	Dp (mm)	p (mm)	Fz_1_ (kN)	Fz_2_ (kN)	Fz Mean (kN)	Standard Deviation	S/N Ratio	Coefficient of Variation
F1	PA	50	6	0.5000	0.6857	0.6806	0.6831	0.0036	3.3099	0.0053
F2	PA	50	8	0.7500	0.9381	0.9348	0.9365	0.0023	0.5703	0.0025
F3	PA	50	10	1.0000	1.2168	1.2178	1.2173	0.0007	−1.7081	0.0006
F4	PA	55	6	0.7500	0.7742	0.7751	0.7746	0.0006	2.2181	0.0008
F5	PA	55	8	1.0000	1.0643	1.0717	1.0680	0.0053	−0.5715	0.0049
F6	PA	55	10	0.5000	0.9321	0.9569	0.9445	0.0176	0.4949	0.0186
F7	PA	60	6	1.0000	0.8565	0.8586	0.8575	0.0015	1.3351	0.0017
F8	PA	60	8	0.5000	0.8276	0.8060	0.8168	0.0153	1.7570	0.0187
F9	PA	60	10	0.7500	1.0995	1.0955	1.0975	0.0028	−0.8081	0.0026
F10	PE	50	6	0.5000	0.4518	0.4225	0.4371	0.0208	7.1825	0.0475
F11	PE	50	8	0.7500	0.6598	0.6554	0.6576	0.0031	3.6409	0.0048
F12	PE	50	10	1.0000	0.9084	0.9459	0.9272	0.0265	0.6552	0.0286
F13	PE	55	6	0.7500	0.4901	0.4937	0.4919	0.0026	6.1623	0.0053
F14	PE	55	8	1.0000	0.6612	0.6645	0.6628	0.0023	3.5718	0.0035
F15	PE	55	10	0.5000	0.6514	0.7126	0.6820	0.0433	3.3155	0.0634
F16	PE	60	6	1.0000	0.5424	0.4969	0.5197	0.0322	5.6772	0.0620
F17	PE	60	8	0.5000	0.5641	0.5700	0.5670	0.0042	4.9278	0.0074
F18	PE	60	10	0.7500	0.7226	0.7391	0.7308	0.0117	2.7235	0.0160

**Table 7:** The results and the statistical processing for the Fx forces.

Cod	Mat.	α (°)	Dp (mm)	p (mm)	Fx_1_ (kN)	Fx_2_ (kN)	Fx Mean (kN)	Standard Deviation	S/N Ratio	Coefficient of Variation
F1	PA	50	6	0.5000	0.3238	0.3183	0.3211	0.0039	9.8674	0.0121
F2	PA	50	8	0.7500	0.4523	0.4496	0.4509	0.0019	6.9181	0.0043
F3	PA	50	10	1.0000	0.5627	0.6189	0.5908	0.0398	4.5613	0.0673
F4	PA	55	6	0.7500	0.4002	0.3974	0.3988	0.0020	7.9853	0.0050
F5	PA	55	8	1.0000	0.5265	0.5313	0.5289	0.0034	5.5321	0.0065
F6	PA	55	10	0.5000	0.4767	0.4667	0.4717	0.0071	6.5262	0.0150
F7	PA	60	6	1.0000	0.4811	0.4836	0.4823	0.0018	6.3332	0.0037
F8	PA	60	8	0.5000	0.3735	0.4258	0.3996	0.0370	7.9479	0.0925
F9	PA	60	10	0.7500	0.5143	0.5915	0.5529	0.0546	5.1263	0.0987
F10	PE	50	6	0.5000	0.2065	0.2067	0.2066	0.0001	13.6975	0.0007
F11	PE	50	8	0.7500	0.3216	0.3287	0.3252	0.0050	9.7578	0.0154
F12	PE	50	10	1.0000	0.4589	0.4524	0.4556	0.0045	6.8273	0.0100
F13	PE	55	6	0.7500	0.2712	0.2736	0.2724	0.0017	11.2965	0.0063
F14	PE	55	8	1.0000	0.3869	0.3830	0.3850	0.0028	8.2911	0.0072
F15	PE	55	10	0.5000	0.3491	0.3500	0.3496	0.0006	9.1298	0.0017
F16	PE	60	6	1.0000	0.3438	0.3444	0.3441	0.0004	9.2655	0.0013
F17	PE	60	8	0.5000	0.2844	0.2822	0.2833	0.0015	10.9544	0.0054
F18	PE	60	10	0.7500	0.4108	0.4073	0.4091	0.0025	7.7641	0.0061

**Table 8:** The results and the statistical processing for the Fy forces.

Cod	Mat.	α (°)	Dp (mm)	p (mm)	Fy_1_ (kN)	Fy_2_ (kN)	Fy Mean (kN)	Standard Deviation	S/N Ratio	Coefficient of Variation
F1	PA	50	6	0.5000	0.3592	0.3089	0.3340	0.0356	9.4993	0.1064
F2	PA	50	8	0.7500	0.4844	0.4263	0.4553	0.0411	6.8159	0.0903
F3	PA	50	10	1.0000	0.5311	0.6373	0.5842	0.0751	4.6330	0.1286
F4	PA	55	6	0.7500	0.3935	0.4084	0.4009	0.0106	7.9370	0.0264
F5	PA	55	8	1.0000	0.5151	0.5408	0.5280	0.0182	5.5454	0.0345
F6	PA	55	10	0.5000	0.4646	0.4785	0.4715	0.0099	6.5285	0.0209
F7	PA	60	6	1.0000	0.4803	0.4832	0.4817	0.0020	6.3441	0.0042
F8	PA	60	8	0.5000	0.3782	0.4430	0.4106	0.0458	7.7050	0.1116
F9	PA	60	10	0.7500	0.4944	0.5997	0.5471	0.0745	5.1993	0.1361
F10	PE	50	6	0.5000	0.2060	0.2062	0.2061	0.0002	13.7193	0.0008
F11	PE	50	8	0.7500	0.2877	0.3663	0.3270	0.0555	9.6468	0.1699
F12	PE	50	10	1.0000	0.4405	0.4737	0.4571	0.0235	6.7941	0.0514
F13	PE	55	6	0.7500	0.2715	0.2721	0.2718	0.0005	11.3140	0.0017
F14	PE	55	8	1.0000	0.3696	0.4127	0.3911	0.0305	8.1406	0.0780
F15	PE	55	10	0.5000	0.3491	0.3512	0.3501	0.0015	9.1154	0.0042
F16	PE	60	6	1.0000	0.3417	0.3461	0.3439	0.0031	9.2719	0.0091
F17	PE	60	8	0.5000	0.2816	0.2848	0.2832	0.0023	10.9581	0.0081
F18	PE	60	10	0.7500	0.4125	0.4083	0.4104	0.0029	7.7351	0.0072

**Table 9:** Response Table for Signal-to-Noise Ratios for the Fz force (with the “smaller is better” condition).

Level	Polymeric Material	Wall Angle	Punch Diameter	Step
1	0.733	2.275	**4.314**	**3.498**
2	**4.271**	2.532	2.316	2.418
3		**2.602**	0.779	1.493
Delta	3.538	0.327	3.535	2.004
Rank	**1**	**4**	**2**	**3**

**Table 10:** Response Table for Signal-to-Noise Ratios for the Fx force (with the “smaller is better” condition).

Level	Polymeric Material	Wall Angle	Punch Diameter	Step
1	6.566	**8.605**	**9.741**	**9.687**
2	**9.665**	8.127	8.234	8.141
3		7.899	6.656	6.802
Delta	3.099	0.706	3.085	2.885
Rank	**1**	**4**	**2**	**3**

**Table 11:** Response Table for Signal-to-Noise Ratios for the Fy force (with the “smaller is better” condition).

Level	Polymeric Material	Wall Angle	Punch Diameter	Step
1	6.575	**8.518**	**9.681**	**9.588**
2	**9.633**	8.097	8.135	8.108
3		7.869	6.668	6.788
Delta	3.058	0.649	3.013	2.799
Rank	**1**	**4**	**2**	**3**

**Table 12:** Analysis of Variance for Means (Fz force).

Source	Degree of Freedom	Adjusted Value of the Sum of the Squares	F Value	*p* Value	Contribution (%)
MAT	1	54.2855	382.8200	0.0000	51.0400
α	2	0.3556	1.2500	0.3510	0.3300
Dp	2	37.7085	132.9600	0.0000	35.4600
s	2	12.0801	42.5900	0.0000	11.3600
MAT*Dp	2	0.9885	3.4900	0.0990	0.9300
MAT*s	2	0.0848	0.3000	0.7520	0.0800
Residual error	6	0.8508			0.8000
Total	17	106.3540			

**Table 13:** Analysis of Variance for Means (Fx force).

Source	Degree of Freedom	Adjusted Value of the Sum of the Squares	F Value	*p* Value	Contribution (%)
MAT	1	38.0950	407.5000	0.0000	40.3000
α	2	1.5591	8.3400	0.0190	1.6500
Dp	2	28.5578	152.7400	0.0000	30.2100
s	2	25.0207	133.8200	0.0000	26.4700
MAT*Dp	2	0.5526	2.9600	0.1280	0.5800
MAT*s	2	0.1841	0.9800	0.4270	0.1900
Residual error	6	0.5609			0.5900
Total	17	94.5302			

**Table 14:** Analysis of Variance for Means (Fy force).

Source	Degree of Freedom	Adjusted Value of the Sum of the Squares	F Value	*p* Value	Contribution (%)
MAT	1	38.9774	440.0500	0.0000	41.9400
α	2	1.3016	7.3500	0.0240	1.4000
Dp	2	27.2472	153.8100	0.0000	29.3100
s	2	23.5355	132.8600	0.0000	25.3200
MAT*Dp	2	0.8809	4.9700	0.0530	0.9500
MAT*s	2	0.4722	2.6700	0.1480	0.5100
Residual error	6	0.5315			0.5700
Total	17	92.9463			

**Table 15:** The coefficients of the regression analysis of the Fz force for polyamide.

Variable	Coefficient	SE Coefficient	T Value	*p* Value
Constant term	0.0740	0.1090	0.6700	0.5300
Wall angle (α)	−0.0022	0.0018	−1.2000	0.2830
Punch diameter (Dp)	0.0787	0.0045	17.4300	0.0000
Step (s)	0.4656	0.0361	12.9000	0.0000

**Table 16:** The coefficients of the regression analysis of the Fz force for polyethylene.

Variable	Coefficient	SE Coefficient	T Value	*p* Value
Constant term	0.2000	0.1740	1.1500	0.3020
Wall angle (α)	−0.0068	0.0029	−2.3800	0.0630
Punch diameter (Dp)	0.0742	0.0072	10.3600	0.0000
Step (s)	0.2823	0.0573	4.9200	0.0040

**Table 17:** The coefficients of the regression analysis of the Fx force for polyamide.

Variable	Coefficient	SE Coefficient	T Value	*p* Value
Constant term	−0.1460	0.0347	−4.2100	0.0080
Wall angle (α)	0.0024	0.0006	4.2000	0.0090
Punch diameter (Dp)	0.0344	0.0014	24.0600	0.0000
Step (s)	0.2731	0.0114	23.8500	0.0000

**Table 18:** The coefficients of the regression analysis of the Fx force for polyethylene.

Variable	Coefficient	SE Coefficient	T Value	*p* Value
Constant term	−0.1867	0.0320	−5.8300	0.0020
Wall angle (α)	0.00164	0.0005	3.1000	0.0270
Punch diameter (Dp)	0.0326	0.0013	24.6600	0.0000
Step (s)	0.2302	0.0106	21.7700	0.0000

**Table 19:** The coefficients of the regression analysis of the Fy force for polyamide.

Variable	Coefficient	SE Coefficient	T Value	*p* Value
Constant term	−0.0987	0.0224	−4.4000	0.0070
Wall angle (α)	0.0022	0.0004	5.9200	0.0020
Punch diameter (Dp)	0.0322	0.0009	34.7300	0.0000
Step (s)	0.2518	0.0074	33.9700	0.0000

**Table 20:** The coefficients of the regression analysis of the Fy force for polyethylene.

Variable	Coefficient	SE Coefficient	T Value	*p* Value
Constant term	−0.1891	0.0238	−7.9600	0.0010
Wall angle (α)	0.0015	0.0004	4.0200	0.0100
Punch diameter (Dp)	0.0330	0.0010	33.6200	0.0000
Step (s)	0.2351	0.0079	29.9500	0.0000

## Data Availability

Not applicable.
